# Left Ventricular Mass in an Oncology Patient and Cocaine User: Metastasis or Thrombus?

**DOI:** 10.1155/cric/3086928

**Published:** 2026-07-11

**Authors:** Dhan Bahadur Shrestha, Prakash Raj Oli, Mustafain Meghani

**Affiliations:** ^1^ Division of Cardiology, Department of Internal Medicine, Bassett Medical Center, Cooperstown, New York, USA, bassett.org; ^2^ Department of Internal Medicine, Mount Sinai Hospital, Chicago, Illinois, USA, hospitalmontesinai.org

**Keywords:** anticoagulation, cocaine-induced cardiomyopathy, intracardiac mass, left ventricular thrombus

## Abstract

**Background:**

Intracardiac thrombus is a common intracardiac mass in patients with left ventricular dysfunction (LVD).

**Case Summary:**

A man with a history of malignant melanoma presented with emesis and acute left flank pain. He was found to have a left renal infarct and left ventricle (LV) mass, for which he was started on therapeutic anticoagulation. TTE showed a large, mobile mass measuring 2.5 × 2.0 cm in the LV cavity, with LVD. Cardiac metastasis was suspected, and surgical intervention was planned, but preoperative TEE could not reveal the LV mass seen previously, canceling the planned thoracotomy.

**Discussion:**

He had patent coronary arteries; therefore, cardiomyopathy was likely due to cocaine use, which might induce thrombosis within the dysfunctional LV. Medical management with therapeutic anticoagulation, along with guideline‐directed medical therapy for LVD, continued.

**Take-Home Messages**

• Left ventricular thrombus (LVT) can mimic intracardiac masses, posing diagnostic challenges; appropriate history, examination, and investigation are key to proper diagnosis and management.
• A multimodality imaging approach and multidisciplinary, shared decision‐making help clinch the diagnosis and avoid unnecessary treatment interventions, such as surgery.
• Cocaine use can manifest with various cardiovascular calamities, including acute coronary syndrome, cardiomyopathy with heart failure, and rarely with LVT due to a combined prothrombotic state and cardiomyopathy.

## 1. Introduction

Cardiac masses are rare yet pose different challenges in their diagnosis and management [1]. The exact prevalence of cardiac masses is unknown, as most of them are discovered incidentally; most of those cardiac masses are tumor‐like conditions, such as thrombus, vegetations, Lambl′s excrescences, or pericardial cysts. After these tumor‐like conditions, secondary cardiac metastatic masses are more common than primary benign and malignant tumors [2]. Intracardiac thrombus is more common on the left side, mostly in the LV, traditionally as a complication of AMI [3]. Here, we present a case of LV thrombus due to cocaine‐related cardiomyopathy and prothrombotic state in the absence of AMI.

## 2. History of Presentation

An adult male in his 50s presented to the emergency department (ED) for nausea, two episodes of nonbloody vomiting, one dark bowel movement, and sudden onset, persistent left flank abdominal pain of 1 h duration. He denied fever, chills, rigors, trauma, fall, urinary frequency, burning micturition, and gross hematuria. Review of systems was unremarkable except for an episode of diminished vision in the left eye, which resolved spontaneously in the morning of the ED presentation. He had a recent ED visit 3 days before his current presentation for generalized abdominal pain and vomiting due to severe constipation.

On arrival, the patient was hemodynamically stable, with a blood pressure of 169/112 mmHg. Physical examination was remarkable for diffuse abdominal tenderness to deep palpation and tenderness at the left costovertebral angle, without guarding or rigidity. In ED, he received intravenous analgesics and antiemetics. ED blood tests were remarkable for neutrophilic leukocytosis (total white blood cell count: 13,800/mm^3^ [normal: 4000–11,000/mm^3^]), and unremarkable urinalysis. High‐sensitivity cardiac troponin was elevated at 303.3 (normal: ≤ 20 pg/mL), but no acute ischemic changes were seen on the electrocardiogram (Figure 1).

**Figure 1:**
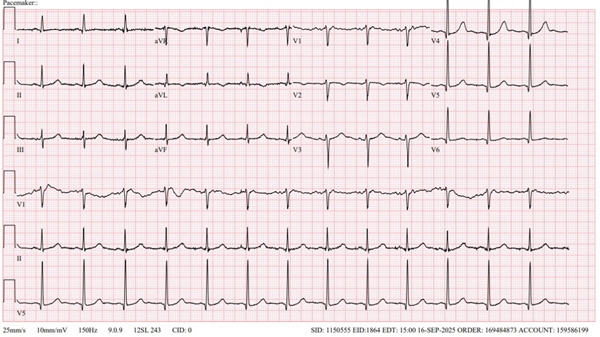
Normal sinus rhythm at 82 bpm, QTc 488 msec, no ST‐T changes suggestive of ischemia.

Computed tomography (CT) of the abdomen and pelvis done in the ED revealed an infarction involving much of the left kidney with patchy enhancement in its mid‐pole, and multiple low‐density filling defects in the left ventricular apex, the largest one measuring 2.9 cm in maximal diameter, as shown in Figure 2.

**Figure 2:**
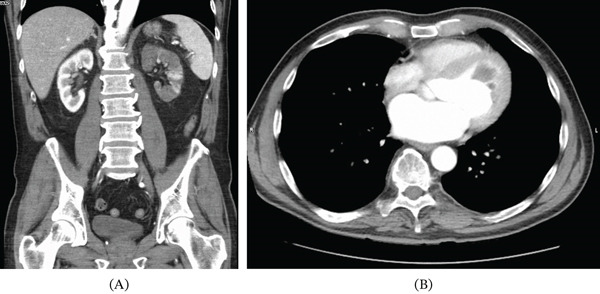
CT abdomen and pelvis showing: (A) near total hypoattenuation of the left kidney with areas of patchy enhancement in the mid‐pole region; (B) hypodense filling defect within the left ventricle.

Initial transthoracic echocardiogram (TTE) showed a large mobile mass likely thrombus in the upper LV cavity measuring 2.5 × 2.0 cm with no visible appendage, moderate left atrial enlargement, and mildly enlarged LV with global hypokinesis with an ejection fraction (EF) of 30% (Figure 3).

**Figure 3:**
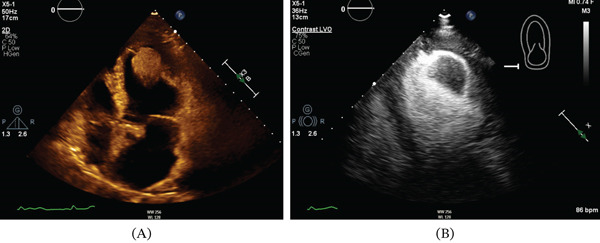
Transthoracic echocardiogram showing: (A) LV mass in apical four‐chamber view; (B) LV mass in apical two‐chamber view with Definity.

## 3. Past Medical History

He had a significant medical history of malignant melanoma of the right shoulder—BRCA+, superficial spreading, pT1a Stage IA, for which he had undergone a wide local excision of the lesion 10 months prior to the presentation. Since the surgical treatment of the melanoma, he has been disease‐free without needing adjuvant chemotherapy, radiotherapy, or immunotherapy. He also had polysubstance use disorder, still using cocaine, and was on a methadone program.

## 4. Differential Diagnosis

As the patient had a significant history of malignant melanoma, a large LV mass, and arterial thrombosis, he was admitted with a working diagnosis of intracardiac thrombus or metastatic melanoma or a combination with systemic thromboembolism. A multidisciplinary discussion was held with the intensive care unit, cardiology, cardiothoracic surgery, and oncology teams, and the patient regarding treatment options, including surgical removal of the mass. A shared decision was made to proceed with surgical resection of the mass, metastatic mass versus thrombus or combination, emergently, given the size of the mass and systemic embolization in the setting of a history of recent melanoma treatment and active cocaine use.

## 5. Management

As soon as the patient was found to have a multifocal new infarction in the left kidney, heparin drip was initiated for anticoagulation following the multidisciplinary discussion with plan of surgical resection of the LV mass, and the patient was admitted to the hospital.

During the second day of admission, a preoperative transesophageal echocardiogram (TEE) performed in the operating room showed a markedly reduced LV mass with some residual anterior layered thrombus between the papillary muscles (Figure 4A).

**Figure 4:**
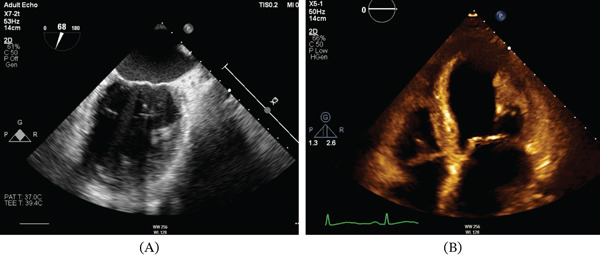
(A) Preoperative TEE showing markedly decreased size of the LV mass with some residual layered thrombus on the papillary muscle; (B) repeat TTE apical four‐chamber view showing markedly decreased size of the LV mass subjacent to the papillary muscle.

This significant reduction in LV mass size prompted the halt of the planned surgical LV mass resection, and the decision was made to continue medical management with therapeutic anticoagulation and serial echocardiographic monitoring of the mass, given the clinical suspicion of an organized thrombus with systemic embolism. Coronary angiogram showed moderate caliber coronary arteries with mild coronary artery disease. Repeat TTE showed a significantly reduced LV mass measuring 1.6 × 0.7 cm, suggestive of thrombus and inferior and lateral wall hypokinesis (Figure 4B). CT angiography of the pulmonary arteries performed on the third day of admission showed an interval decrease in LV filling defects, with no pulmonary embolism.

## 6. Outcome and Follow‐Up

On the fourth day of admission, he developed a sudden onset of bilateral foot pain, was able to ambulate, but had sharp pain in the bilateral lower extremities with pins and needles sensation in his feet. Physical examination revealed the right foot was cold and pale, whereas the left foot was warm with quick capillary refill. Doppler showed a weak, sporadic signal on the right foot and a strong signal on the left. CT angiogram aorta run‐off showed thrombus in the right tibio‐peroneal trunk and left popliteal artery with poor opacification of the calf arteries distally. Patient was managed medically, after multidisciplinary discussion with the vascular surgery team, with continued therapeutic anticoagulation with heparin. For the asymptomatic left ventricular systolic dysfunction due to non‐ischemic cardiomyopathy (NICMP) and possible cocaine‐induced cardiomyopathy, he was started on guideline‐directed medical therapy (GDMT). On the 10th day of admission, he was transitioned to apixaban for a duration of at least 6 months. He was counseled in detail about quitting substance abuse, particularly cocaine use, and a psychology referral was sent for cognitive behavioral therapy for cocaine use disorder.

Patient is seen and followed by a multidisciplinary team in the outpatient setting, has completed a 3‐month follow‐up, and is doing well; has also completed follow‐up imaging surveillance with magnetic resonance imaging (MRI) of the brain and positron emission tomography (PET) tumor imaging. His follow‐up MRI brain 2 months after admission did not show any acute intracranial process or metastasis. The PET scan also did not show any fluorodeoxyglucose (FDG) activity suspicious for metastasis.

## 7. Discussion

Cardiac masses are rare yet increasingly diagnosed more than they used to be a decade back, with the advancement in noninvasive imaging technology as well as their wider application. Despite these advancements in diagnostic modalities, accurate diagnosis and, consequently, appropriate management remain complex clinical challenges. The correct diagnosis of the cardiac masses is utmost, as mostly they are pseudo‐tumors such as thrombus, vegetations, Lambl′s excrescences, which are often misdiagnosed, resulting in significant morbidity, sometimes mortality to patients, mainly from unnecessary treatment or interventions. Thus, there has been an evolving concept of multimodality imaging approach in the evaluation of complex and heterogeneous cardiac masses [1].

Intracardiac thrombus has been one of the common causes of intracardiac masses, primarily involving the LV. LV thrombus is a dreadful complication of LV dysfunction, which poses the high risk of subsequent stroke and systemic embolism, morbidity, and mortality [3, 4]. LV dysfunction leading to LV thrombus occurs in the majority due to ischemic cardiomyopathy (ICMP), most often from AMI, whereas the remaining cases are due to NICMP, such as dilated cardiomyopathy from varied etiologies like genetic mutations/infections/infiltrative/toxins, takotsubo cardiomyopathy, LV noncompaction cardiomyopathy, perinatal cardiomyopathy, and cardiac amyloidosis [4]. In our case, the patient is not aware of any kind of cardiac diseases in his personal as well as other family members, and has denied any sudden deaths among first/second degree relatives. The only significant personal history was cocaine use.

Recreational substances abuse, mainly cocaine, have been well established as a risk factor for various forms of cardiomyopathies and cocaine‐related ICMP with LV thrombus. Lee et al. reported a 34‐year‐old patient with large LV thrombus secondary to cocaine‐induced cardiomyopathy [5]. Similar to our case, Fortier et al. reported a case of apical LV thrombus with peripheral embolization to the right lower extremity in the setting of cocaine‐induced AMI, but in contrast to our case, this patient required surgical thrombectomy of the LV thrombus [6].

The pathophysiology of LV thrombus involves the well‐established mechanism of Virchow′s triad, an interplay of: [1] stasis attributable to reduced left ventricular function, [2] ventricular wall tissue damage, and [3] inflammation/hypercoagulability [4, 7]. In our case, he had a significant history of chronic as well as recent cocaine use, 2 days prior to the current presentation. Cocaine is well‐known to have a diverse pathophysiological effect, leading to adverse cardiovascular events. It causes persistent overstimulation of the sympathetic system by inhibiting catecholamine reuptake at sympathetic nerve terminals, thereby increasing the sensitivity of adrenergic nerve endings to norepinephrine. Its acute use often causes an abrupt surge in catecholamines, leading to endothelial dysfunction, coronary vasoconstriction via inhibition of nitric oxide (NO) synthase, increased endothelin‐1 levels, altered intracellular calcium handling, and vessel injury from acutely elevated blood pressure. Chronic use of cocaine might lead to cardiomyopathy via different mechanisms: ischemic by inducing myocardial ischemia or infarction, or nonischemic via adrenergic surge and thus the above‐mentioned molecular imbalance, chronic cardiomyocyte exposure to elevated norepinephrine leading to cardiomyocyte apoptosis via *β*‐receptor and superoxide dismutase activation, myocyte volume increase, persistent oxidative stress state by inducing the reactive oxygen species formation, and/or causing the eosinophilic myocarditis [4, 7, 8]. Cocaine also has prothrombotic effects by increasing the platelet activity and aggregation, elevating the levels of fibrinogen and von Willebrand factor, and increasing the activity of the plasminogen activator inhibitor [8]. Our case had mild coronary disease on coronary angiography but regional wall motion abnormalities with reduced LVEF due to chronic cocaine exposure, possible endothelial dysfunction, and prothrombotic state following the acute cocaine exposure, thus setting Virchow′s triad in play, finally resulting in the formation of the LVT, which had systemic embolic shower in the form of left renal thrombosis and bilateral lower extremity arterial emboli.

As mentioned, the diagnosis of LV thrombus must be made using a multimodality imaging approach that includes echocardiography (transthoracic and/or transesophageal), cardiac magnetic resonance imaging (CMR), and/or CT [4]. TTE is the first‐line imaging modality for diagnosing LV thrombus and assessing its presence, shape, and size. It has high sensitivity and specificity for LV thrombus, but it is highly operator dependent, and its sensitivity may be doubled with the use of an ultrasound‐enhancing agent during echocardiography. LV mass must be well‐defined, an echogenic intracavitary mass with clear demarcation from the surrounding ventricular cavity and no endocardial attachment and visualized throughout the cardiac cycle. TEE is generally not recommended as a secondary imaging modality because it has limited ability to visualize the LV apex, where most LV thrombus forms. But sometimes TEE can provide additional evidence to distinguish LV thrombus from other cardiac masses, especially if performed before surgical intervention, as in our case, and thus help guide appropriate management changes that may prevent unnecessary intervention, particularly a surgical one. Cardiac CT scan has limited value in diagnosing LV thrombus and has comparable sensitivity and specificity to TTE.

CMR is considered the gold standard in evaluation of any cardiac mass following its echocardiographic assessment, as it offers higher sensitivity and specificity than echocardiogram. It provides a comprehensive assessment of location, morphology, surrounding tissue relationships, hemodynamic effects, and tissue characterization. Its sensitivity and specificity are further enhanced by late gadolinium enhancement, which produces higher‐resolution anatomic images. CMR can distinguish benign from malignant pathologies with diagnostic accuracy ranging from 92% to 100% and can characterize LV thrombus, which is devoid of vascularity and resembles the surrounding ventricular wall [1]. CMR also plays a pivotal role for assessing ventricular size, function, and tissue in dilated cardiomyopathy patients. LGE and T1 mapping detect different types of fibrosis and provide prognostic insights into remodeling, arrhythmias, therapy response, and sudden cardiac death beyond traditional measures such as EF [9]. If cardiac CT or CMR remains inclusive in differentiating the malignancy nature of intracardiac mass, then ^18^F‐FDG PET/CT can be useful at the cost of suboptimal specificity. It also for staging of the malignancy and detecting the primary of the cardiac metastasis [1].

The recommended multimodality imaging approach is TTE with contrast, followed by MRI if TTE remains inconclusive, or CT with contrast if MRI is unavailable or not tolerated [4, 7]. In our case, our multispecialty rural hospital has a dedicated CMR only once a week, with one MRI scanner dedicated to cardiac scans. We have limitations due to our limited scanner and the cardiologist reading the MRI; therefore, we could not obtain an MRI, and after shared decision‐making, we planned to proceed emergently with surgical resection of the mass/thrombus, given its size.

Management of LV thrombus must be multidisciplinary, involve shared decision‐making with the patient, and may include medical management without surgical intervention. For LV thrombus in the setting of NICMP, the 2022 AHA scientific statement recommends oral anticoagulation (direct oral anticoagulant or warfarin) for 3‐6 months, with discontinuation if LVEF improves to >35% (assuming resolution of the LV thrombus) or if major bleeding occurs. They do not suggest the use of fibrinolytic therapy for the primary purpose of LV thrombus treatment. Surgical resection should be reserved for those patients who do not tolerate anticoagulation or are deemed to be at high risk of embolization or cardioembolic stroke despite anticoagulation [7]. Prolonged anticoagulation and repeated cardiac imaging (TTE) should be considered for patients with protruding, mobile thrombus until its resolution, given their higher risk of embolization compared with immobile, calcified, or organized thrombus [4]. Our patient has a mobile LV thrombus and reduced LVEF, so we decided to continue anticoagulation with apixaban for at least 6 months and repeat surveillance TTE to confirm complete thrombus resolution.

## 8. Conclusion

Cardiac masses, despite their rarity, have been increasing with advances in and utilization of noninvasive cardiac imaging. They pose complex clinical scenarios that require accurate diagnosis and appropriate management, where proper history, including substance abuse, can be crucial information. Any cardiac masses must be evaluated using a multimodality imaging approach, and multidisciplinary shared decision‐making should guide their management to avoid unnecessary interventions, such as surgery, since most are tumor‐like pathologies, for example, thrombus.

**Nomenclature**
AMI: acute myocardial infarction
CT: computed tomography
GDMT: guideline‐directed medical therapy
LV: left ventricle
LVD: left ventricular dysfunction
MRI: magnetic resonance imaging
NICMP: nonischemic cardiomyopathy
TTE: transthoracic echocardiogram
TEE: transesophageal echocardiogram

## Author Contributions

P.R.O., D.B.S., and M.M. were involved in the patient′s clinical care and management, contributed to the literature review, and assisted in drafting and revising the manuscript. P.R.O. contributed substantially to the manuscript′s conception and preparation, conducted the literature review, and was primarily responsible for drafting and critically revising it. D.S. supervised the overall project, contributed to the clinical oversight and intellectual content, and was involved in the critical revision of the manuscript. D.S. acted as guarantor and is responsible for the overall integrity of the content of the manuscript.

## Funding

No funding was received for this manuscript.

## Disclosure

All authors have read and approved the final version of the manuscript and agree to be accountable for all aspects of the work in accordance with ICMJE authorship criteria.

## Ethics Statement

Given that this is a case report and written informed consent was obtained from the patient, ethical approval is not required.

## Consent

Written informed consent has been obtained from the patient for publication of this case report and any accompanying images.

## Conflicts of Interest

None of the authors have a conflict of interest to disclose.

## Data Availability

The data that support the findings of this study are available on request from the corresponding author. The data are not publicly available due to privacy or ethical restrictions.

## References

[1] Angeli F. , Bodega F. , Bergamaschi L. , Armillotta M. , Amicone S. , Canton L. , Fedele D. , Suma N. , Cavallo D. , Foà A. , Belmonte M. , Russo V. , Attinà D. , Niro F. , Bonfiglioli R. , Fanti S. , Pavon A. G. , Guglielmo M. , Mushtaq S. , Pantaleo M. A. , Andreini D. , Lovato L. , Pontone G. , Lopez-Mattei J. , Paolisso P. , and Pizzi C. , Multimodality Imaging in the Diagnostic Work-Up of Patients With Cardiac Masses: JACC: CardioOncology State-of-the-Art Review, JACC: CardioOncology. (2024) 6, no. 6, 847–862, 10.1016/j.jaccao.2024.09.006, 39801632. 39801632 PMC11711820

[2] Tyebally S. , Chen D. , Bhattacharyya S. , Mughrabi A. , Hussain Z. , Manisty C. , Westwood M. , Ghosh A. K. , and Guha A. , Cardiac Tumors: JACC CardioOncology State-of-the-Art Review, JACC: CardioOncology. (2020) 2, no. 2, 293–311, 10.1016/j.jaccao.2020.05.009, 34396236. 34396236 PMC8352246

[3] McCarthy C. P. , Murphy S. , Venkateswaran R. V. , Singh A. , Chang L. L. , Joice M. G. , Rivero J. M. , Vaduganathan M. , Januzzi J. L. , and Bhatt D. L. , Left Ventricular Thrombus, Journal of the American College of Cardiology. (2019) 73, no. 15, 2007–2009, 10.1016/j.jacc.2019.01.031. 30846340

[4] Zhang Q. , Zheng H. , Zhang Z. , Xu Y. , and Zhang W. , Advancing Clinical Management Of Left Ventricular Thrombosis: Prevention, Detection And Treatment Modalities In The Modern Era, Heart. (2025) 111, no. 14, 662–670, 10.1136/heartjnl-2024-324605, 39938942. 39938942 PMC12229065

[5] Lee S. N. , Zadikany R. H. , Thomson L. E. J. , Siegel R. J. , and Berman D. S. , Cigar-Shaped Left Ventricular Thrombus in Cocaine-Related Cardiomyopathy: Echocardiography, Cardiac MRI and Coronary CT Angiography Correlations, European Heart Journal - Cardiovascular Imaging. (2024) 25, no. 12, e326–e326, 10.1093/ehjci/jeae220, 39162028. 39162028

[6] Fortier S. , Demaria R. G. , Pelletier G. B. , Carrier M. , and Perrault L. P. , Left Ventricular Thrombectomy in a Cocaine User With Normal Coronary Arteries, Journal of Thoracic and Cardiovascular Surgery. (2003) 125, no. 1, 204–205, 10.1067/mtc.2003.118, 12539009. 12539009

[7] Levine G. N. , McEvoy J. W. , Fang J. C. , Ibeh C. , McCarthy C. , Misra A. , Shah Z. I. , Shenoy C. , Spinler S. A. , Vallurupalli S. , Lip G. Y. H. , and American Heart Association Council on Clinical Cardiology; Council on Cardiovascular and Stroke Nursing; and Stroke Council , Management of Patients at Risk for and With Left Ventricular Thrombus: A Scientific Statement From the American Heart Association, Circulation. (2022) 146, no. 15, E205–E223, 10.1161/CIR.0000000000001092, 36106537. 36106537

[8] Havakuk O. , Rezkalla S. H. , and Kloner R. A. , The Cardiovascular Effects of Cocaine, Journal of the American College of Cardiology. (2017) 70, no. 1, 101–113, 10.1016/j.jacc.2017.05.014. 28662796

[9] Patel A. R. and Kramer C. M. , Role of Cardiac Magnetic Resonance in the Diagnosis and Prognosis of Nonischemic Cardiomyopathy, JACC: Cardiovascular Imaging. (2017) 10, 1180–1193, 10.1016/j.jcmg.2017.08.005, 28982571. 28982571 PMC5708889

